# Impact of Patient Blood Management on Red Blood Cell Utilization in an Urban Community Teaching Hospital: A Seven-Year Retrospective Study

**DOI:** 10.3390/life14020232

**Published:** 2024-02-07

**Authors:** Ding Wen Wu, Mark T. Friedman, Daniel P. Lombardi, Richard Hwang, Joel Sender, Valdet Cobaj, Masooma Niazi, Yanhua Li, Robert Karpinos

**Affiliations:** 1 Department of Pathology, SBH Health System, Bronx, NY 10457, USA; dingwen.wu@nyulangone.org (D.W.W.); vcobaj@sbhny.org (V.C.); mniazi@sbhny.org (M.N.); 2 Department of Pathology, New York University Grossman School of Medicine, New York, NY 10016, USA; 3 Department of Pathology, New York University Grossman Long Island School of Medicine, Mineola, NY 11501, USA; 4 Department of Medicine, SBH Health System, Bronx, NY 10457, USA; dlombardi@sbhny.org (D.P.L.); senderj@outlook.com (J.S.); 5 Division of Transfusion Medicine, Department of Medicine, UMass Chan Medical School, Worcester, MA 01655, USA; yanhua.li@umassmemorial.org; 6 Department of Anesthesiology, SBH Health System, Bronx, NY 10457, USA; rkarpinos@sbhny.org

**Keywords:** patient blood management, red blood cell, transfusion, hemoglobin, retrospective study

## Abstract

Objectives: This study aimed to showcase how implementing a patient blood management (PBM) program effectively cuts unnecessary red blood cell (RBC) transfusions in a New York City urban community teaching hospital. Methods: Analyzing seven years from 2013 to 2019, a retrospective review of RBC transfusions was conducted. Results: Following the introduction of PBM, considerable improvements were observed annually. These included a drop in mean pretransfusion hemoglobin levels from 7.26 g/dL (2013) to 6.58 g/dL (2019), a 34% reduction in yearly RBC unit transfusions, and fewer units given to patients with pre-Hgb levels ≥ 7 g/dL (from 1210 units in 2013 to 310 units in 2019). Furthermore, this study noted a decline in two-unit RBC orders when Hgb levels were ≥ 7 g/dL from 65 orders in 2013 to merely 3 in 2019. The estimated total cost savings attributed to the six-year PBM program duration after full implementation in 2014 amounted to USD 2.1 million. Conclusions: Overall, PBM implementation significantly decreased RBC transfusions and enhanced transfusion practices. The findings emphasize that successful PBM strategies do not always necessitate extensive resources or increased budgets but instead rely on the application of intuitive methods, as evidenced by this study.

## 1. Introduction

Introducing patient blood management (PBM) principles in a small hospital, despite the absence of a specific budget allocation, poses challenges. However, various strategic steps can be pursued to integrate PBM practices without relying on additional funding.

A 350-bed urban community teaching hospital serves an acute care trauma center in a sub-served area of New York City. The hospital provides a comprehensive array of residency training programs, covering specialties such as psychiatry, dentistry, surgery, podiatry, pediatrics, emergency medicine, osteopathic manipulative medicine, neuromusculoskeletal medicine, internal medicine, and dermatology.

Using an organized, evidence-based multidisciplinary approach [1] to transform blood transfusion practices to optimize patient care for the patients who might need a blood transfusion, a patient blood management (PBM) program was initiated in 2013 and fully implemented in 2014 at the hospital. 

The PBM program was designed to promote better blood management practices, reduce transfusion rates, and improve patient outcomes. To achieve these goals, the implementation of the program required the cooperation of all healthcare professionals involved in the transfusion process, including physicians, nurses, laboratory technicians, and administrators.

One of our key strategies employed was the use of broad-based educational modules [2] for licensed practitioners. These modules were updated and provided annual education, especially for new attending physicians, residents, fellows, physician assistants, and nurse practitioners. The education was designed to improve practitioners’ understanding of blood management principles and to promote best practices for the use of blood products. Transfusion guidelines and clinical transfusion protocols were adjusted to focus on symptomatic anemia rather than on hemoglobin triggers. This approach reduced the use of blood products in cases where transfusions were not clinically indicated. 

Another critical aspect of the PBM program was the conversion of laboratory samples to low-volume testing. By using smaller samples, the laboratory was able to perform more efficient and cost-effective testing, reducing the need for unnecessary blood draws and minimizing the risk of iatrogenic anemia.

Furthermore, the program included a reduction in the critical-level reporting of hemoglobin (Hgb) from 7 g/dL to 6 g/dL. This change helped ensure that transfusions were only given when necessary, based on the patient’s symptoms and clinical condition. 

Finally, the initial orderable number of red blood cell (RBC) units in the electronic medical health record system was set to one unit instead of two units [3]. This change helped to reduce the number of unnecessary RBC transfusions and decreased the overall cost of the transfusion process.

Overall, the PBM program was effectively implemented without any specifically allocated budgetary support. The program’s success was due in large part to the collaboration and dedication of all healthcare professionals involved in the transfusion process, as well as the commitment to evidence-based best practices for blood management. 

## 2. Materials and Methods

We conducted a retrospective analysis of all RBC transfusions from 2013 to 2019 to examine the impact of this PBM program on RBC utilization in the hospital. 

This retrospective study was given exempt status by the institutional review boards of St. Barnabas Health. 

### 2.1. Hypothesis

The implementation of an evidence-based PBM program would effectively reduce unnecessary RBC transfusions in the hospital.

#### 2.1.1. Aim

The goal of this study is to analyze the impact of this PBM program. 

#### 2.1.2. Data Selection Criteria

We retrospectively analyzed our health system’s blood transfusion database from 1 January 2013 to 31 December 2019. RBC transfusion events without exclusion for all patients admitted during this seven-year period were identified, regardless of age, gender, race, or disease condition. However, certain exclusions were applied, such as emergency transfusion events involving the use of uncrossmatched RBC for trauma and acute severe bleeding cases, as well as events following the massive transfusion protocol. Outpatient transfusion events were excluded. Transfusion events with plasma products, platelet products, or cryoprecipitate were also excluded since this PBM study only focused on inpatient RBC transfusions.

#### 2.1.3. Study Endpoints

To examine the impact of this restrictive PBM program, the following endpoints from 2014 to 2019 were analyzed and compared with baseline data from 2013:

1. Average (and median) of pre-transfusion Hgb levels that triggered the RBC transfusion orders.
2. Volume of RBC transfusion units.
3. Annual volume of RBC transfusion units when the pre-transfusion Hgb level was ≥ 7 g/dL (thereafter referred to as # Hb le7).
4. Annual percentage of RBC transfusion units when the pre-transfusion Hgb level was ≥ 7 g/dL (thereafter referred to as % Hb le7).

This comparison was used to show the reduction, if any, of unnecessary RBC transfusions since, in the context of the PBM program, orders for RBC transfusions in nonbleeding patients with Hgb ≥ 7 g/dL without symptomatic anemia or known cardiac disease are unnecessary. Thus, the impact on # Hb le7 and % Hb le7 reflect the effect on the reduction of unnecessary RBC transfusions.

5. The number of two-unit RBC transfusion orders.
6. When Hgb ≥ 7 g/dL, the number and percentage of two-unit orders of RBC transfusion.
7. Overall rate of RBC transfusions.

To remove the variations caused by the patient length of stay (LOS) and the number of admissions/discharges for the comparison, the annual overall rate of RBC transfusion is calculated and compared to the one in 2013.

The overall rate of RBC transfusions is calculated as follows: Overall rate of RBC transfusion = RBC transfusions without exclusions per 1000 patient days. Patient days = total number of discharges X average LOS (days).

8. Length of stay (LOS).

Since the goal of PBM is to optimize care for patients who might need a blood transfusion, the impact estimation was not limited to transfused patients only. The yearly LOS for all of the inpatients in 2014–2019 was analyzed and compared with the LOS for 2013.

9. Potential cost savings from reducing unnecessary RBC transfusions.
  This estimation is based on a reduction of % Hb le7.
  Calculation steps (see Table 1):
  (1) Number of transfused RBC units.
  (2) Number of RBC units transfused when Hgb triggers ≥ 7 g/dL.
  (3) % of RBC units transfused when Hgb trigger ≥ 7 g/dL.
  (4) Potential reduction of RBC units = ((3) of year 2013 (3) of year of interest (yi)) × (1) of yi.
  (5) Potential cost savings of yi = (4) potential reduced RBC units of yi × USD 1000/unit.

Per estimation [4], it costs about ~USD 1000 per unit of RBC transfusion. 

The total potential cost saving of years 2014–2019 = the summary of potential cost saving from years 2014 to 2019.

#### 2.1.4. Statistics

Quantitative data were expressed as the arithmetic mean ± standard deviation. The median of each group’s data was also sometimes displayed as desired. Statistical analysis was conducted with the unpaired t-test using Excel 2019 (Microsoft Corp., Redmond, WA, USA) to evaluate the difference between each endpoint value mentioned above and the counterpart value for the year 2013. Values of *p* < 0.05 were considered statistically significant. 

## 3. Results

**Figure 1:**
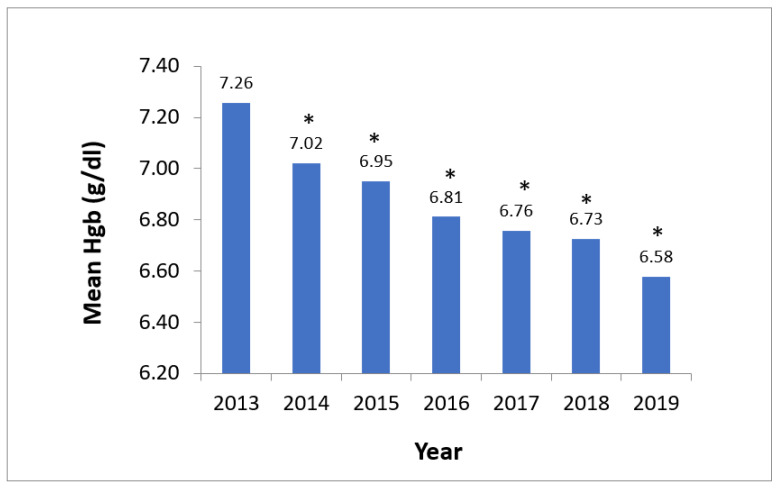
Average hemoglobin level for RBC transfusion orders. Note: * The *p*-value is < 0.0001 for the value of each year from 2014 to 2019 compared to the number for the year 2013.

**Figure 2:**
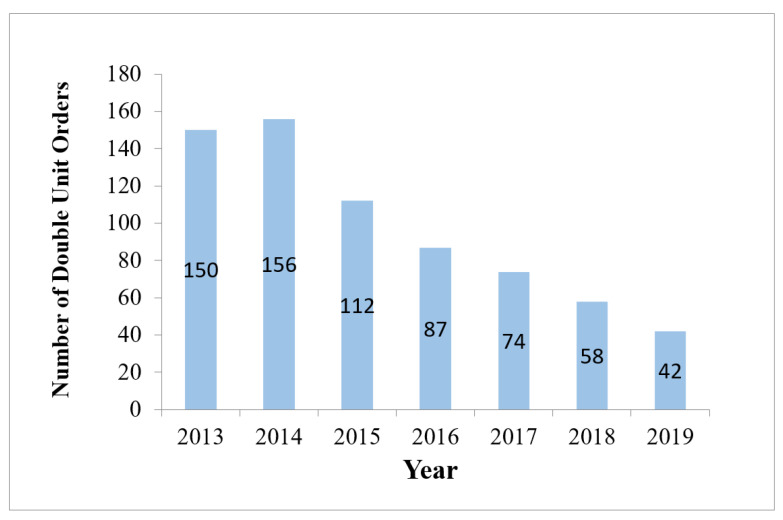
Number of double RBC orders.

**Figure 3:**
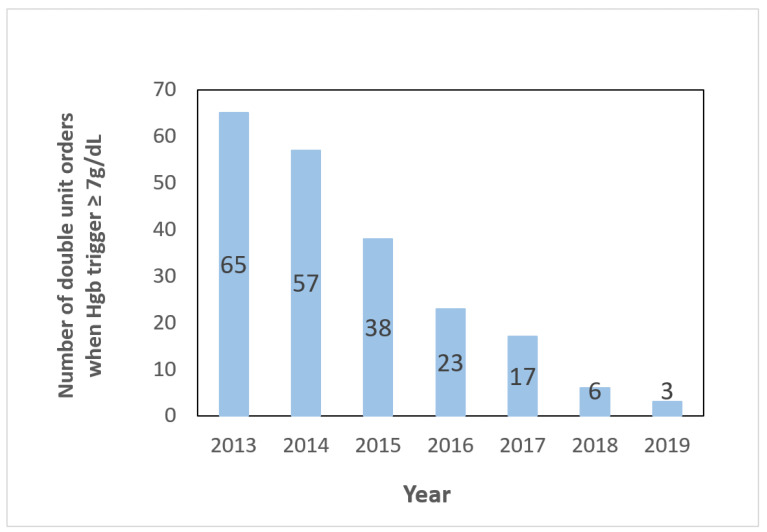
Number of double RBC orders when Hgb ≥ 7 g/dL.

**Figure 4:**
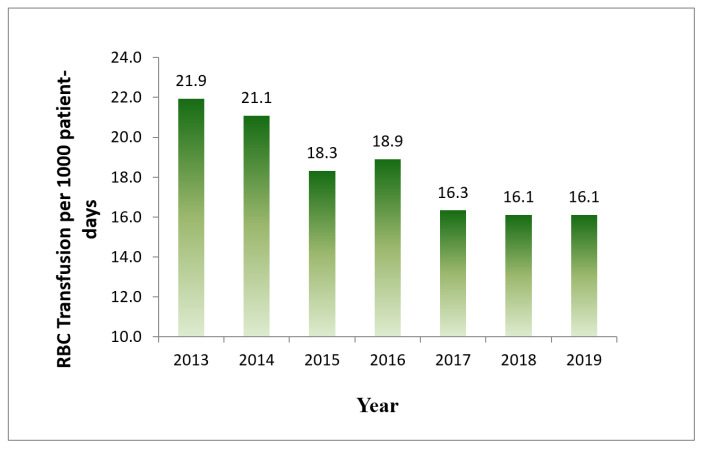
Annual inpatient RBC transfusion rate.

Our results show that significant improvements in RBC transfusion practices and usage have occurred since the implementation of the PBM program in 2014, as follows:

- Mean pre-transfusion Hgb for RBC transfusion orders consistently decreased year-over-year after PBM program implementation, comparing a nadir of 6.58 g/dL in 2019 to 7.26 g/dL in 2013 (*p* < 0.0001) (Figure 1).

- Median Hgb for RBC transfusion orders revealed the same trend (Supplementary Data Figure S1).
- During the same six-year period (2014–2019), the number of annual transfused RBC units showed a significant decrease of 34% compared to the one in 2013 (Table 1; (2061–1350) ÷ 2061 = 34%).
- Similarly, the absolute number and percentage of transfused RBC units with Hgb trigger ≥ 7 g/dL significantly decreased year-over-year, reaching a nadir in 2019 compared to 2013 (310 units vs. 1210 units, respectively, *p* < 0.0001, and 23.0% vs. 58.7%, respectively) (Table 1). This reflects a significant reduction (by 35.7%) of unnecessary RBC transfusions consistently year after year. 58.7% − 23.0% = 35.7%.
- The number of two-unit RBC transfusion orders decreased from 150 to 42 post-intervention (Figure 2).

- When Hgb trigger ≥ 7 g/dL, the number and percentage of two-unit orders for RBC transfusions decreased from 65 (3.4% of RBC transfusions) to 3 (0.2%) post-intervention (*p* < 0.0001), as shown in Figure 3.

- The annual overall rate of RBC transfusion without exclusion per 1000 patient days decreased from 21.9 in 2013 to 16.1 in 2019 (Figure 4), demonstrating a 26% reduction.

Overall rate of RBC transfusions = units of inpatient RBC transfusion without exclusions per 1000 inpatient days.

- Minimal change in the mean and median inpatient LOS over seven years suggested no hospital patient harm caused by the restrictive PBM program (Table 2).

- This RBC usage reduction translates into approximately 2115 units of RBC saved during the six-year post-launch period (2014–2019). Based on a cost of ~USD 1000 per unit [4], the potential cost savings peaked at ~USD 482,000 in 2019, with total savings of ~USD 2.1 million during the six years of PBM implementation (Table 1).
- The data presented in this paper are original.

Remarkably, the advancement of this PBM program was achieved without any additional budget allocated to it.

## 4. Discussion

The scope of overtransfusion and the problems associated with it are significant in healthcare. Overtransfusion refers to the unnecessary or excessive administration of blood or blood products to patients. Some key issues associated with overtransfusion [5,6] are as follows:

- Transfusion reactions and complications: Receiving unnecessary blood transfusions increases the risk of adverse reactions, which can range from mild to severe transfusion-associated circulatory overload (TACO), transfusion-related acute lung injury (TRALI), allergic reactions, hemolytic reactions, and transfusion-transmitted infections, among others.
- Potential for adverse outcomes: Overtransfusion may not improve patient outcomes and can potentially lead to increased morbidity and mortality in certain cases [6].
- Increased healthcare costs: Blood transfusions are costly and can substantially contribute to healthcare expenses. Unnecessary transfusions lead to increased healthcare spending without providing commensurate benefits to the patient.
- Resource utilization: Inappropriate blood use leads to a strain on blood bank resources, potentially resulting in shortages for patients who genuinely require transfusions.

Shander et al. illustrated that the key strategies in PBM include support of hematopoiesis and improving hemoglobin level, optimizing coagulation and hemostasis, use of interdisciplinary blood conservation modalities, and patient-centered decision making throughout the course of care [1].

Patient blood management (PBM) aims to mitigate these problems by optimizing blood use, minimizing unnecessary transfusions, and improving patient outcomes through evidence-based practices tailored to individual patient needs. It emphasizes a personalized approach to care that prioritizes patient safety and well-being while conserving precious blood resources [1,7,8,9]. 

The primary pillars of PBM typically include:

(1) Optimizing red blood cell mass: Ensuring patients have adequate red blood cell levels before surgery or invasive procedures through treatments like iron supplementation, erythropoietin therapy, or other medications. (2) Minimizing blood loss: Employing surgical techniques, such as minimally invasive surgery, to reduce blood loss during procedures. Additionally, use specific medications or interventions to control bleeding. (3) Enhancing patients’ tolerance to anemia: Some patients may tolerate lower blood levels without experiencing adverse effects. PBM involves identifying patients who can safely function with lower hemoglobin levels without the need for a transfusion. (4) Appropriate blood transfusion: Making evidence-based decisions on when to transfuse blood and ensuring that transfusions are only administered when necessary and beneficial.

Implementing PBM in a small hospital without a dedicated budget can be challenging, but there are several steps that can be taken to introduce PBM principles [5,6] without requiring significant additional funding.

The subsequent table encapsulates diverse approaches for executing PBM without the need for supplementary financial backing (Table 3). 

Chau et al. reported that a successful multidisciplinary PBM program involved a series of educational lectures, consultations, and discussions with doctors, nurses, and theater staff [2]. 

By focusing on education, utilizing existing resources efficiently, implementing evidence-based guidelines, and fostering an interdisciplinary culture of responsible blood utilization, a hospital can implement PBM practices without a dedicated budget.

Our study validates the principle that successful implementation of practical PBM strategies is achievable without budgetary support and no funds allocated toward the PBM program in a small urban community teaching hospital. Main outcome measures across the seven-year study period showed that statistically significant (i.e., *p* < 0.05) improvements in RBC utilization occurred marked by reductions in the overall number of transfused RBC units, transfusion rate per 1000 patient days, two vs. single unit transfusions, transfusions with a Hgb trigger greater than 7 g/dL, and blood product wastage. In addition, PBM implementation led to significant cost savings. These beneficial study outcomes were realized despite the inclusion of all inpatient populations, including trauma and neonatal patients; larger reductions in blood utilization and cost savings may have resulted had these patient populations been excluded from the study. The observational nature of this study is a noted limitation, though.

Jenkins et al. implemented a restrictive transfusion strategy with a typical quality improvement framework and, based on education and computerized physician order entry (CPOE) enhancements, significantly reduced off-protocol and total blood transfusions [7]. Our keys to successful implementation included ongoing PBM education via online learning modules targeted for licensed practitioners, along with the use of PBM tools such as restrictive transfusion guidelines, computerized physician order entry alerts with clinical decision choices in the hospital EMR system to guide physicians in transfusion decisions, the use of low-volume test sample tubes to reduce hospital-acquired anemia, and the reduction in the critical Hgb alert value from 7 g/dL to 6 g/dL. The calculation method of the overall RBC transfusion rate by Jenkins et al. was utilized in this study. 

In comparison, Warner et al. published observational data from an eight-year study to support the successful implementation of PBM in a large U.S. academic medical center without evidence of patient harm. Their data, which included 400,998 admissions, showed a 33% reduction of allogenic transfusions per 1000 admissions, an absolute risk reduction for transfusion of 6%, and a 22% decrease in the rate of transfusions over projected [8].

PBM has emerged as a crucial aspect of healthcare, seeking to optimize patient outcomes, enhance safety, and achieve cost savings. In the study conducted by Hofmann et al., the implementation of PBM was explored across 12 nations through in-depth, semi-structured interviews. The findings revealed a unanimous consensus among healthcare professionals on the potential benefits of PBM, including improved patient outcomes, enhanced safety measures, and financial savings. However, despite this agreement, several barriers to successful PBM implementation were identified. One of the primary obstacles highlighted by this study was the limited experience with PBM among healthcare practitioners. The lack of familiarity with PBM practices posed a significant challenge, emphasizing the need for comprehensive education and training initiatives. Additionally, this study identified the necessity for a shift in work practices and emphasized the crucial roles of collaboration and communication in facilitating successful PBM integration. To address these challenges and enhance the implementation of PBM, this study proposed six intervention levels. These levels encompassed various stakeholders, including government entities, healthcare providers, educational institutions, funders, research organizations, and patients [9]. 

Recognizing the significance of education in fostering better patient care, the focus of our PBM project was on educating healthcare providers. In alignment with the findings of the broader study, the PBM project underscored the importance of healthcare providers adopting dual roles. Providers were encouraged not only to serve as front-line care providers but also as leaders in quality improvement initiatives. This approach aimed to empower healthcare professionals with the knowledge and skills needed to effectively implement and practice PBM within their clinical settings. By fostering a culture of continuous learning and quality improvement, the PBM project sought to overcome barriers to implementation. The dual role of healthcare providers as both caregivers and quality improvement leaders was envisioned as a pivotal strategy for creating sustainable change. Through education, training, and the cultivation of leadership skills among healthcare providers, the PBM project aimed to contribute to broader efforts to enhance patient care, safety, and the overall effectiveness of healthcare delivery. 

The “Why give 2 when 1 will do?” Choosing Wisely campaign, inspired by Podlasek et al. and Warner et al. [3,10], further exemplifies the commitment to sustaining positive changes. We launched a “Why give 2 when 1 will do?” Choosing Wisely campaign, and we have been sustaining it using education and computer-physician-order entry alerts with clinical decision support (CPOE-CDS). We witnessed an overall decrease of 2-unit orders for Hgb ≥ 7 g/dL progressively from 65 orders per year (3.4% of total orders) to 3 orders (0.2%) from 2013 to 2019. 

Fischer et al. studied the effect of PBM implementation on physicians’ risk perception, clinical knowledge, and perioperative practice across four German university hospitals. Key implementation strategies targeted knowledge (i.e., development and distribution of PBM educational materials and local guidelines; standardization of performance metrics and data collection to allow valid benchmarking within organizations, etc.); attitude (i.e., fostering team spirit and corporate identity; aggressive marketing, etc.); and behavior (i.e., changing the infrastructure and preoperative flow of patients; preoperative anemia assessment; measurement of individual physician transfusion practice, etc.). Utilizing pre- and post-implementation questionnaires, Fischer et al. demonstrated significant positive shifts in physicians’ attitudes toward preoperative anemia treatment and patient assessment after each single-unit RBC transfusion. Moreover, there was a noteworthy reduction in the percentage of physicians routinely using Hgb < 6 g/dL as an indicator for transfusion. This reduction was coupled with an increase in those favoring physiological transfusion triggers, such as electrocardiogram changes or lactic acidosis [11].

In parallel, the strategies implemented in response to the “Why give 2 when 1 will do?” Choosing Wisely campaign aligns with Fischer et al.’s findings. The educational efforts and the integration of CPOE local guidance in the original text are reflected in the success story of improved clinicians’ knowledge, attitude, and behavior regarding RBC transfusion orders. The progressive and substantial decrease in RBC transfusions observed further reinforces the positive impact of these combined strategies. These parallel successes underscore the universality of certain principles in optimizing blood transfusion practices. Both the campaign and Fischer et al.’s study emphasize the importance of education, attitude change, and behavioral modification in achieving positive outcomes in the realm of patient blood management. The amalgamation of these principles, as evident in the original text, stands as a testament to the multifaceted and comprehensive approach needed to bring about meaningful change in healthcare practices. 

Our strategies of education and CPOE local guidance successfully improved clinicians’ knowledge, attitude, and behavior toward RBC transfusion orders, with evidence of a progressively substantial decrease in RBC transfusions.

Finally, in its policy brief calling for the urgent implementation of PBM, the World Health Organization [12] highlighted data from the largest study on PBM outcomes to date published by the Western Australia PBM Program. This study included over 600,000 patients admitted to Western Australia’s four major adult hospitals during a six-year study period and showed significant reductions in mortality, infection, acute myocardial infarction and stroke, and hospital length of stay. Improved key indicators showed reductions in preoperative anemia, pre-transfusion Hgb, and transfused blood products. Meanwhile, single-unit RBC transfusions increased. This program resulted in an estimated product cost savings of USD 18.5 million and activity cost savings of USD 80–USD 100 million [13]. Our study demonstrated that our PBM was successful in reducing unnecessary blood products with a total cost savings of 2.1 million US dollars over 6 years (2014–2019). Our experience also showed that the PBM can be achieved in a community hospital without budgetary support. Lowering the hemoglobin trigger for blood transfusion may reduce the amount of blood transfused to patients, which could result in lower risks of transfusion reactions, infections, and other adverse events associated with blood transfusions. On the other hand, a lower hemoglobin trigger may increase the risk of patient harm, especially in patients with cardiac disease or other medical conditions where a higher hemoglobin level may be required to maintain adequate oxygen delivery. The decision to lower the hemoglobin trigger from 7 g/dL to 6 g/dL for non-cardiac patients was made in conjunction with an interdisciplinary team of healthcare providers, taking into account the patient’s individual needs, the clinical context, and the best available evidence. 

The minimal change in the mean and no change in the median of the inpatient length of stay over a 7-year period indicates that the restrictive PBM program did not result in harm to hospital patients.

Another limitation in our PBM implementation is that although the initial orderable RBC units were set to one unit per order in our EMR, the full impact of computerized physician order entry with clinical decision support (CPOE-CDS) may not have been realized to its maximum potential. Goodnough and Hollenhorst [14] demonstrated a 42% reduction in RBC transfusions using a targeted CPOE-CDS to promote restrictive blood transfusion practices in their institution through smart best practice alerts (SBPA’s) that are triggered for RBC orders above 7 g/dL or 8 g/dL for patients with acute coronary syndrome or post-cardiothoracic procedure. The inclusion of CPOE-CDS with SBPA’s could potentially have impacted further reductions in RBC transfusions in our institution.

The study authors believe that this report demonstrates that successful PBM can be achieved without the allocation of significant additional resources or budgetary support through the implementation of a few common-sense strategies. It should serve as a source of inspiration for other healthcare facilities that have yet to embark on their journey toward successful PBM implementation. 

To further enhance the impact of PBM, future directions should focus on continuous improvement and adaptation to evolving healthcare landscapes. This includes ongoing education and training programs, updates to clinical guidelines based on emerging evidence, using targeted CPOE-CDS with smart best practice alerts to promote restrictive blood transfusion practices, and the integration of advanced technologies such as artificial intelligence to optimize decision-making processes.

Continuous monitoring and evaluation of PBM initiatives are essential for identifying areas for improvement and ensuring sustained positive outcomes. Collaboration with other healthcare institutions and participation in national and international initiatives can facilitate the sharing of best practices, fostering a global community committed to advancing patient blood management.

## 5. Conclusions

This retrospective seven-year study demonstrated that this multidiscipline PBM program in an urban community teaching hospital using education and restrictive tools effectively reduced total RBC usage without specific budgetary support. In particular, the PBM program markedly reduced the unnecessary RBC transfusion for Hgb ≥ 7 g/dL (by 35.7%) and total RBC transfusion (by 28%) and reduced unnecessary 2-unit RBC orders for Hgb ≥ 7 g/dL (from 3.4% to 0.2%). The potential cost savings is estimated at 2.1 million US dollars. This research underscores how effective PBM implementation can occur without the need for substantial extra resources or increased budgetary support by adopting a handful of practical strategies.

## Figures and Tables

**Table 1:** RBC units transfused and potential cost savings.

Year	2013	2014	2015	2016	2017	2018	2019	Total
(1) Number of transfused RBC units	2061	1762 *	1460 *	1514 *	1251 *	1286 *	1350 *	
(2) Number of RBC units transfused when Hgb trigger ≥ 7 g/dL	1210	830 *	572 *	517 *	371 *	357 *	310 *	
(3) % of RBC units transfused when Hgb trigger ≥ 7 g/dL = (2) ÷ (1)	58.7	47.1	39.2	34.1	30.0	27.8	23.0	
(4) Potential reduction of RBC units = ((3) of year 2013 − (3) of this year) × (1) of the year) **	0	204	285	372	375	397	482	2115
(5) Potential cost saving (USD) = (4) × USD 1000/ unit	0	204K	285K	372K	375K	398K	482K	USD 2.1 million

* *p*-value < 0.0001 compared to the number in year 2013. ** Row (4) calculation example: potential reduction in RBC units in 2014 = (58.7% − 47.1%) × 1762 = 204 units.

**Table 2:** Hospital inpatients’ length of stay (LOS).

LOS (Days)	2013	2014	2015	2016	2017	2018	2019
Mean (Days)	5.1	4.9	5.5	5.5	5.2	5.3	5.5
Standard deviation (Days)	13.1	9.3	12.5	8.7	10.8	10.5	11.2
Median (Days)	3.0	3.0	3.0	3.0	3.0	3.0	3.0

**Table 3:** Ways to Introduce PBM without Additional Funding.

Education and training	Start by educating staff, including physicians, nurses, and other healthcare professionals, about the principles and benefits of PBM. Utilize internal expertise that may offer free or low-cost educational materials and training sessions.
Clinical guidelines and pathways	Develop and implement evidence-based clinical guidelines and pathways that emphasize conservative blood management strategies. These guidelines can include practices such as minimizing unnecessary blood tests, optimizing hemoglobin levels pre-operatively, and employing blood conservation techniques during surgery.
Utilize existing resources	Assess and optimize the use of existing resources within the hospital. Work with the laboratory and clinical staff to reduce unnecessary blood tests, adopt restrictive transfusion thresholds, and explore alternatives to transfusions, such as iron supplementation or medications that reduce bleeding.
Quality improvement initiatives	Implement quality improvement initiatives aimed at reducing blood product waste, improving blood utilization practices, and ensuring that transfusions are given based on the established clinical criteria rather than routine practice.
Collaboration and partnerships	Collaborate with blood banks, regional blood centers, or other healthcare facilities in the area to explore cost-sharing opportunities, joint training programs, or information sharing related to best practices in blood management.
Utilize data and analytics	Leverage data analytics to monitor blood utilization patterns, transfusion rates, and associated costs. This information can help identify areas for improvement and guide decision making to optimize blood utilization without requiring additional funds.
Engage stakeholders	Involve stakeholders across different departments and specialties in discussions about PBM. Encourage collaborative efforts to implement changes and promote a culture of responsible blood use throughout the hospital.
Adopt technology solutions	Implement electronic health record (EHR) systems or clinical decision support tools that can help clinicians adhere to evidence-based transfusion guidelines, thereby reducing unnecessary blood transfusions.
Evaluate and Adjust	Continuously monitor the impact of PBM initiatives on patient outcomes, transfusion rates, and associated costs. Use these data to refine strategies and make the necessary adjustments to improve the effectiveness of PBM practices.
Seek Grants or collaborative opportunities	Look for grants or collaborative opportunities within the healthcare community that support PBM initiatives or projects aimed at improving blood management. Participating in such programs can provide additional resources and support without requiring a separate budget allocation.

## Data Availability

Data are unavailable due to privacy and ethical restrictions.

## Supplementary Materials

The following are available online at https://www.mdpi.com/article/10.3390/life14020232/s1, Figure S1: Median hemoglobin level for RBC transfusion orders * *p* value < 0.0001 comparing to the number in year 2013.
