# Supplementary material for: Impact of Patient Blood Management on Red Blood Cell Utilization in an Urban Community Teaching Hospital: A Seven-Year Retrospective Study
Source: Life (Basel). 2024 Feb 7;14(2):232. doi: 10.3390/life14020232 (PMC10890656; doi:10.3390/life14020232)
Supplement: Supplementary file 1 [file life-14-00232-s001.zip › life-2844879-SI.pdf]

Supplementary Materials

Impact of Patient Blood Management on Red Blood Cell Utilization in an Urban Community Teaching Hospital: A Seven-Year Retrospective Study

Ding Wen Wu [1,2], Mark T. Friedman [3,*], Daniel P. Lombardi [4], Richard Hwang [1], Joel Sender [4], Valdet Cobaj [1], Masooma Niazi [1], Yanhua Li [5] and Robert Karpinos [6]

[1] Department of Pathology, SBH Health System, Bronx, NY, 10457, USA; dingwen.wu@nyulangone.org (D.W.W.); vcobaj@sbhny.org (V.C.); mniazi@sbhny.org (M.N.)
[2] Department of Pathology, New York University Grossman School of Medicine, New York, NY, 10016, USA
[3] Department of Pathology, New York University Grossman Long Island School of Medicine, Mineola, NY, 11501, USA
[4] Department of Medicine, SBH Health System, Bronx, NY, USA; dlombardi@sbhny.org (D.P.L.); senderj@outlook.com (J.S.);
[5] Division of Transfusion Medicine, Department of Medicine, UMass Chan Medical School, Worcester, MA, USA; yanhua.li@umassmemorial.org
[6] Department of Anesthesiology, SBH Health System, Bronx, NY, USA; rkarpinos@sbhny.org
* Correspondence: mark.friedman@nyulangone.org; Tel.: +1-516-663-8307; Fax: +1-516-663-4581

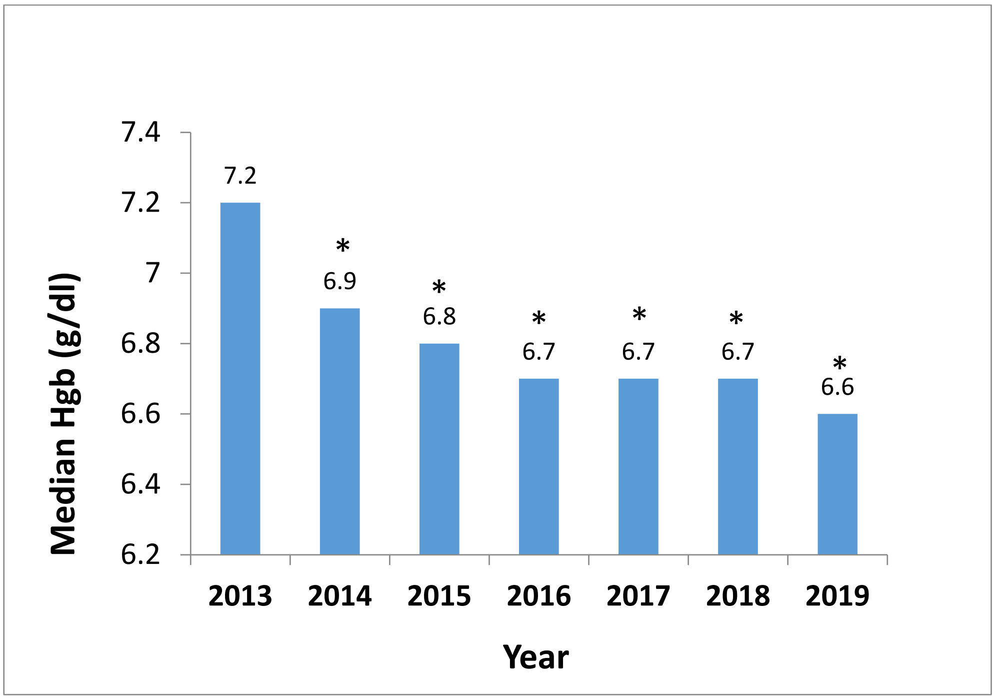

Figure S1. Median hemoglobin level for RBC transfusion orders * P value < 0.0001 comparing to the number in year 2013.
